# Non-Invasive Diagnostic Techniques in Dermatology

**DOI:** 10.3390/jcm12031081

**Published:** 2023-01-30

**Authors:** Victor Desmond Mandel, Marco Ardigò

**Affiliations:** Porphyria and Rare Diseases Unit, San Gallicano Dermatological Institute—IRCCS, Via Elio Chianesi 53, 00144 Rome, Italy

The search to enhance the clinical diagnostic accuracy for identifying skin cancer has led to the development of non-invasive diagnostic techniques in dermatology including dermoscopy, reflectance confocal microscopy (RCM) and optical coherence tomography (OCT) [1]. These tools offer useful information for a rapid non-invasive diagnosis and for the identification of a biopsy site, which can aid pathology evaluation and save time ahead of a confirmatory diagnosis, anticipating treatment and improving patient management.

Dermoscopy is a straightforward tool to evaluate the patient because it utilizes a ×10 to ×100 microscope objective with a light source to magnify and visualize structures present below the skin’s surface. This method, routinely used in adjunct to clinic evaluation, is helpful as the first approach to many diseases, especially in the field of dermato-oncology where it improves the diagnosis of melanoma at an earlier stage [2], non-melanoma skin cancers [3], and other rare skin tumours such as cutaneous T-cell lymphomas [4] and Kaposi’s sarcoma [5]. However, dermoscopy is also associated with unnecessary skin biopsy or excision of benign lesions and a risk of misdiagnosing. Among the non-invasive skin imaging devices, in vivo RCM allows for the visualization of the epidermis, dermoepidermal junction and papillary dermis at a quasi-histological resolution, providing horizontal grayscale colour images related to the refractive index of different tissues and cell structures [6]. RCM has been demonstrated to be very useful for the diagnosis of basal cell carcinoma and its subtyping through the identification of specific confocal criteria [7]. A randomized clinical trial proved that adjunctive use of RCM for suspect melanocytic lesions reduces unnecessary excisions and assures the removal of aggressive melanomas at baseline [8]. RCM can also be helpful in making the distinction between a precancerous lesion and squamous cell carcinoma, especially in body sites such as the lip where the epidermis is thinner [9]. OCT, on the other hand, combines principles of ultrasonography and optical interferometry to provide real-time structural details of both horizontal and vertical sections of the skin [6]. Moreover, dynamic OCT (D-OCT) enables the additional evaluation of vascular details of the tissue [1]. OCT shows a higher penetration depth (up to 2 mm) in comparison to RCM (about 200 µm), but a lower resolution. This technique is extremely useful for the diagnosis of actinic keratosis and non-melanoma skin cancer, especially in cases of hyperkeratosis and/or the presence of ulceration, which are well-known factors that may limit the effectiveness of RCM [6]. D-OCT can identify specific microvascular features that allow us to distinguish between nevi and melanoma, overcoming OCT’s inability to distinguish melanocytic lesions [10].

However, non-invasive diagnostic techniques are also increasingly applied in other fields of dermatology. These technology tools may also assist in the diagnosis of inflammatory and autoimmune skin diseases that can be grouped together, based on histopathologic features, as psoriasiform, spongiotic and interface dermatitis, bullous diseases, and scleroderma [1]. Moreover, they can be used to detect precise microscopic features of acne elementary lesions, allowing accurate quantifications for disease severity staging and guiding the clinician in the prescription of tailored treatment protocols based on each patient’s characteristics [11]. In the trichology field, the synergic use of non-invasive technology tools is very useful for the diagnosis of the different alopecia types and their therapeutic monitoring, providing an excellent assessment of morphological changes occurring in the scalp area during treatment and during long-term follow-up [12]. The nail apparatus consists of highly structured, large anatomical units of varying optical densities, and may, therefore, be particularly suitable for imaging. Dermoscopy allows for observation of the nail surface at a high magnification, whereas RCM and OCT can measure the thickness, density and roughness, providing a new and objective evaluation of the nail unit [13].

Non-invasive diagnostic techniques can also be used to monitor wound healing, separating the findings into the three stages of wound healing (inflammatory, proliferative and remodelling phase) [14]. Another application of these technologies is the skin ageing assessment. Clinical manifestations of ageing correlate with the skin cytoarchitectural background detectable with RCM and OCT [15].

In conclusion, non-invasive diagnostic techniques were proven to be useful in diagnosis, follow-up and assessing treatment response, avoiding repeated biopsies and identifying the best target area for biopsy.
